# The impact of telemedicine enabled pre-hospital triage in acute stroke – a protocol for a mixed methods systematic review

**DOI:** 10.12688/hrbopenres.13514.2

**Published:** 2023-10-30

**Authors:** Deirdre McCartan, Stuart Lee, Jorin Bejleri, Paul Murphy, Anne Hickey, David Williams

**Affiliations:** 1 iPASTAR Collaborative Doctoral Award Programme, Division of Population Health Sciences, Division of Population Health Sciences, RCSI University of Medicine and Health Sciences, Dublin, Ireland; 2 School of Medicine, RCSI University of Medicine and Health Sciences, Dublin, Ireland; 3 Stroke Medicine/Gerontology, Beaumont Hospital, Dublin, Ireland; 4 RCSI Library, Royal College of Surgeons in Ireland, Dublin, Ireland; 5 Department of Psychology, RCSI University of Medicine and Health Sciences, Dublin, Ireland

**Keywords:** Acute stroke, thrombolysis, thrombectomy, primary stroke centre (PSC), comprehensive stroke centre (CSC), prehospital, telemedicine, healthcare systems

## Abstract

**Introduction:**

Increasing access to thrombolysis and thrombectomy through improved pathway organisation remains a health service challenge that requires contextualisation to the geographic, demographic and resourcing status of any regional stroke service. Pre-hospital delays or delays during inter-hospital transfers can result in patients being outside the window for one or both interventions. Pre-hospital triage using technology-enabled interdisciplinary communication networks may facilitate rapid individualized care decisions, permitting streamlined care pathways to hospital sites most appropriate to their clinical presentation and history in the first instance. Understanding the experience of those involved in efforts to improve or reorganise care may help to explain the impact observed.

**Objectives:**

1. To review the impact of pre-hospital telemedicine enabled workflow intervention strategies on patient outcomes and on service process metrics in hyper-acute stroke care

2. To examine how the experience of those involved in providing or receiving such interventions might identify key characteristics of effective interventions

**Inclusion criteria:**

Quantitative, qualitative and primary mixed methods studies will be included. Quantitative studies will assess effectiveness of telemedicine-enabled interventions that facilitate pre-hospital acute stroke triage. Intervention effects on functional outcomes of patients, on intervention rates and on key time metrics in hyperacute stroke care will be assessed. Qualitative studies will explore the experiences of people involved in or impacted by these interventions.

**Methods and analysis:**

A convergent segregated mixed methods systematic review will synthesise and integrate primary qualitative, quantitative and mixed methods studies using the Joanna Briggs Institute methodology. Database searches will include OVID (MEDLINE), EMBASE, The Cochrane Library, CINAHL and Web of Science. Critical appraisal will include the Mixed Methods Assessment Tool. Results of quantitative studies and findings of qualitative studies will be integrated and configured to explore and contextualize each single method synthesis.

**Systematic review registration:**

This protocol has been submitted for registration with PROSPERO.

## Introduction

Intravenous thrombolysis (IVT) and endovascular thrombectomy (EVT) are time-dependent revascularisation treatments used in the very early (<24 hours since symptom onset) phase of acute stroke that can result in significantly improved patient outcomes, with greatest effect achieved the earlier they are delivered ^1, 2^. Parallel improvements in telecommunications infrastructure and reliability, with increasing evidence for highly effective interventions for hyperacute stroke care, have led clinicians and researchers to consider information and communications technology (ICT) based approaches to address treatment delays in the provision of acute stroke care. Telemedicine is defined by the World Health Organisation as “the provision of healthcare services at a distance with communication conducted between healthcare providers seeking clinical guidance and support from other healthcare providers (provider-to-provider telemedicine); or conducted between remote healthcare users seeking health services and healthcare providers (client-to-provider telemedicine)” ^3^. Applying ICT solutions to health system challenges offers potential ways to maximise access to limited human and infrastructure resources. Telemedicine has been shown to reduce death and dependency when applied to stroke unit care ^4^. Understanding the role of telemedicine systems in acute stroke care is a research priority outlined in The Stroke Action Plan for Europe 2018–2030 (SAP-E) ^5^. However, while ICT solutions can act as a platform to enable improved human performance they do not replace it. Telemedicine solutions hinge on human factors to maximise their effectiveness including buy-in for piloting and implementation.

To understand the challenges of systems change we can learn from translational research which acknowledges that human behaviour takes place in complex social situations and that knowledge and intention do not necessarily result in predictable, expected or presumed behaviour. Woolf described how Type 2 translational research is concerned with *“*human behavior and organizational inertia, infrastructure and resource constraints, and the messiness of proving the effectiveness of “moving targets” under conditions that investigators cannot fully control” ^6^. This review will address these challenges by using a mixed methods approach to understanding the experiential aspects involved in effective telemedicine interventions used in the hyperacute stroke care context.

Quantitative studies such as randomized control trials give us reliable evidence of effect but do not explain context – the why, the where and for whom an intervention is effective. Qualitative research can offer insights into how those involved in or affected by an intervention or system change experience or perceive it, including potentially unintended or unforeseen impacts or consequences. Mixed methods review approaches have been used to better understand the impact of interventions aimed at improving outcomes in chronic disease management ^7^. This review seeks to synthesise the existing evidence from both quantitative and qualitative research paradigms to identify effective telecommunications strategies for patient triage in the pre-hospital phase of acute stroke, and to identify and understand key characteristics of effective strategies through the experiences of participants.

## Protocol

### Objective

To summarise and synthesise existing qualitative and quantitative evidence of the impact of telemedicine on key patient and service level outcomes in acute stroke, when used to facilitate patient triage in the pre-hospital setting, and to understand how human factors impact on these complex health system interventions in the acute stroke pathway.

### Review questions

**Quantitative**: What is the impact of pre-hospital telemedicine enabled triage on functional outcomes of patients experiencing acute stroke, and on key time metrics reflective of quality in hyperacute stroke care?

**Qualitative**: How do the experiences and perspectives of end users of telemedicine-based interventions help explain the effectiveness, acceptability and feasibility of such interventions?

## Methods

A convergent segregated mixed method systematic review (MMSR) will be conducted in line with the Joanna Briggs Institute (JBI) methodology ^8^.

This protocol has been guided using the PRISMA-P checklist ^9^. The protocol has been submitted for prospective registration on the international database for systematic reviews, PROSPERO. This protocol establishes a priori the authors’ intentions for this MMSR.

The writing and reporting of the review will be guided by the Preferred Reporting Items for Systematic Reviews and Meta-Analysis (PRISMA) 2020 statement and the Enhancing Transparency in Reporting the Synthesis of Qualitative research (ENTREQ) guidelines ^10, 11^.

This mixed method systematic review (MMSR) will include quantitative, qualitative and mixed method studies.

### Study eligibility

**Study design:** This mixed method systematic review will assess telemedicine enabled communication strategies or networks that facilitate and support pre-hospital decision making on the most appropriate patient disposition or routing (primary stroke centre or comprehensive stroke centre) of patients experiencing acute stroke when compared to usual care. Usual care is defined as undifferentiated transportation of patients to their local hospital.

Primary quantitative studies will include randomised controlled trials (RCTs), non-randomised controlled trails (NRCTs), controlled before-after studies (CBA) and interrupted time series and repeated measures studies (ITS). We will also include retrospective or prospective observational cohort studies.

Qualitative studies will include any primary research study that uses recognised methodologies for data collection and analysis. Data collection techniques may include interviews, focus group discussions or surveys, while standard accepted qualitative analysis and synthesis methods such as narrative, thematic or framework approaches may be included. Feasibility studies or process evaluations that include formal qualitative methodology for data collection and analysis will also be included.

Primary mixed methods studies which permit disaggregation and analysis of quantitative and qualitative data separately will be included. If the quantitative or qualitative component of any primary mixed methods study does not meet the pre-established criteria above then only the study component (quantitative or qualitative) meeting the criteria will be included with the second study component data excluded at the full text screening step.

### Quantitative inclusion criteria

Using the following population, intervention, comparison and outcome (PICO) elements for quantitative studies or quantitative components of mixed methods studies we will identify studies meeting our inclusion criteria:

**Population:** The target study population will be adults ≥ 18yrs with suspected acute stroke <24hrs since symptom onset, unknown time of symptom onset or wake-up stroke (WUS). (WUS is defined here as the scenario where a person wakes up with symptoms of stroke having gone to bed previously symptom free, and where their last known well time >4.5 hours prior ^12^).

**Intervention:** The quantitative component of this review will consider studies that examine the use of telemedicine-enabled interventions for patient triage in the pre-hospital setting of acute stroke care. Such interventions should aim to facilitate rapid communication, decision making and triage of patients to the most appropriate destination stroke centre (PSC or CSC) potentially bypassing the geographically nearest hospital, in order to take a person directly to a stroke centre deemed most suitable to their clinical presentation.

Telemedicine in this context may comprise: telephone communications between the pre-hospital and hospital setting that also include a formal objective clinical assessment tool; televisual or tele-audio-visual communications including smartphone applications or other remote Wi-Fi-enabled communication links to hardware on board an ambulance to the hospital-based team; Mobile Stroke Units (MSU). (While MSUs may be deemed an interventional service for the provision of rapid thrombolysis, it may also be considered a rapid triage tool for those who could benefit from thrombolysis and/or thrombectomy). 

Included studies may focus on IVT, EVT or both. Any transportation mode will be considered. Pilot studies that meet the inclusion criteria will be included. 

This review is focused on pre-hospital tele-triage and so will not include studies that use telemedicine to facilitate remote hospital-based clinical assessment (hub-and-spoke models) or to facilitate inter-hospital transfer decisions between a PSC and CSC (drip-and-ship models).

It is acknowledged that some studies will include results for all patients evaluated by a triage intervention while other studies will report results only of patients receiving treatment for acute ischaemic stroke (eg. onset to needle time, door to needle time, door to puncture time), some of whom will include stroke mimics. If a study does not report its rate of treatment of mimics, authors will be contacted for more specific treatment metrics related to mimics. If additional information on the treatment rate of mimics is not available this will be highlighted and the study excluded where deemed appropriate.

**Comparison:** The quantitative component of the review will consider studies that compare the intervention to the immediate transportation of a patient with stroke or stroke-like symptoms to the nearest hospital without pre-hospital differentiation or triage (usual care). Historical controls will be accepted. A control group must be present for any quantitative study to be included.

**Outcomes:** The quantitative component of this review will consider studies that include the following outcome measures: the primary patient-centred outcome will be assessment of functional ability at 90 days using the Modified Rankin Scale (mRS). Safety will be assessed using the 90-day mortality rate.

Secondary quantitative outcomes will be considered at a health service level, where available, to include the key time metrics, proportions of people receiving IVT and additional clinical impacts. The following outcomes will be considered: symptom onset to hospital (door) time; onset to decision time thrombolysis (IVT); onset to decision time endovascular thrombectomy (EVT); onset to treatment time (“onset to needle” for (IVT) and “onset to puncture” for (EVT)); alarm to treatment times; door to first imaging time; overall rates of interventions (IVT and EVT); rates of interventions <60 minutes from symptom onset; time to first contact with CSC; NIHSS at 24 hours; rate of symptomatic intracranial haemorrhage at <1 week; proportion of stroke mimics treated with IVT; hospital length of stay; ambulance usage time. Additionally, depression, cognition and quality of life at 90 days will be assessed where recorded.

### Qualitative inclusion criteria

Using the following Population, Phenomenon, Context (PICo) for qualitative studies, or the qualitative components of mixed methods studies, we will identify studies meeting the following inclusion criteria:

**Population:** Any adult ≥18 years involved in the provision or receipt of an intervention as described above. This may include patients, family members or healthcare staff.

**Phenomenon of Interest:** The qualitative component of this review will consider studies that investigate the experiences, perspectives and perceptions of those involved in providing or receiving a telemedicine-based intervention.

**Context:** The setting of the qualitative studies will primarily be in the pre-hospital phase of the acute stroke pathway may cross both primary and secondary care.

### Exclusion criteria

We will exclude studies involving children or young people ≤17yr. Studies that involve inter-hospital transfers without pre-hospital triage or studies that used patient simulations will be excluded. Studies that use ICT for pre-notification of ambulance arrival purposes only will not be sufficient for inclusion. We will exclude systematic reviews or any trial without a control.

**Search strategy:** A systematic search of electronic databases MEDLINE (Ovid), EMBASE, The Cochrane Library, CINAHL and Web of Science, will be undertaken using a search strategy based on the key concepts under review developed in collaboration with a data information specialist(PM). A preliminary scoping search identified qualitative and quantitative studies of interest in the area. An expanded search using relevant terms including stroke, telemedicine, pre-hospital and triage will be incorporated into medical subject headings, and key words will be searched and combined systematically in an iterative approach to maximise information retrieval. No language or date limits will be applied. A sample search strategy for MEDLINE (Ovid) is provided in Table 1 and will be adapted for all databases as appropriate. Published studies in any language available in full text that can be reliably translated into English will be included. Reference lists of included full text articles will be further screened for relevant studies.

**Table 1.** Search strategy for MEDLINE (Ovid).

	OVID MEDLINE ALL April 2022
1	exp Stroke/ or exp Ischemic Stroke/ or stroke.mp. or cerebral hemorrhage.mp. or exp Cerebrovascular Disorders/ or *Brain Ischemia/
2	Exp Ambulances/ OR emergency medical service$.mp. or *Emergency Medical Services/ OR (time adj2 treatment).ti,ab. or exp Time-to-Treatment/ OR (pre-hospital adj2 treatment) or (prehospital adj2 treatment) OR *Triage/ OR (onset adj2 door) OR (door adj2 treatment) OR (door adj2 reperfusion) OR (door adj2 needle ) OR (door adj2 groin)
3	(mobile adj1 stroke) OR mobile stroke unit$.mp. OR hospital bypass.mp. OR exp telemedicine/ OR telemedicine.mp. OR telestroke.mp. OR exp Remote Consultation/
**4 **	**1 AND 2 AND 3 **

**Study selection:** Two researchers (DMcC and SL) will independently screen by title and abstract against the documented eligibility criteria using Covidence software ^13^. A solution to any disagreements will initially be through conferral and discussion between the two reviewers. Unresolved disagreements will be discussed with the project supervisors (DW and AH) in order to reach consensus.

Included studies will be retrieved in full text and will again be screened independently by the two reviewers. Disagreement on inclusion of any study at this stage will be resolved by discussion, or again with the project supervisors, if necessary to gain consensus. Reasons will be recorded for studies excluded at full-text stage. A PRISMA flow diagram will record all steps for accuracy and transparency.

**Data management:** A record will be maintained of all final database searches. References will be managed using EndNote20.1. Screening software will permit efficient screening of titles and abstracts, and of full texts, independently by both reviewers. Review Manager (RevMan) version 5.4 will be used for meta-analysis and qualitative synthesis if results permit through its open-access link to GRADE-Pro software.

**Data extraction:** A data collection form will be designed to ensure that complete and consistent data extraction from quantitative, qualitative and mixed methods studies is achieved. Collected data will include year of publication, study design, study setting, population or perspective, participant numbers and demographics, intervention, control or comparator, primary outcome, secondary outcomes, measures of intervention effect and findings or themes.

Reviewers will be trained on the utilization of the data collection form and it will be piloted prior to use to ensure utility and consistent use by reviewers. Data extraction will be undertaken independently by the same reviewers involved in screening. Disagreement on inclusion of any data will be resolved by discussion as described for screening steps. Efforts will be made to find missing data including by contacting study authors.

**Assessment of methodological quality**: The Mixed Methods Appraisal Tool (MMAT) will used at study level to assess methodological quality of empirical quantitative (including RCTs, non-randomised studies and quantitative descriptive studies), qualitative and mixed methods studies, using five domains for each design assessed ^14^. Judgements of quality will be by two reviewers undertaking independent appraisals. Sensitivity analyses will be undertaken where possible and appropriate, excluding any studies of low methodological quality if they exert any undue influence on estimates. Unpublished data will be included in this review if it made available by authors. Should none be made available, this limitation will be acknowledged when interpreting any funnel plot assessing potential publication bias.

The TIDIER (Template for Intervention Description and Replication) checklist will be used as a guide to reporting of details of included interventions.

**Overall assessment of certainty:** An assessment of the overall certainty in the accumulated evidence will be made using the GRADE and Grade-CerQual tools for quantitative and qualitative studies respectively that permits an evidence to decision framework. GRADE narrative statements will further clarify the authors’ confidence in the evidence extracted in the review.

**Data synthesis and integration:** The convergent segregated mixed methods approach to this systematic review involves separate but parallel synthesis of quantitative results and qualitative findings. Data will not be transformed from quantitative to qualitative or vice versa and a clear distinction will be maintained between quantitative results (including the quantitative results of MM studies) and qualitative results (including the qualitative results of MM studies) until the integration step. Synthesis of each single method type will be complete before moving to integration. Quantitative results will be synthesised using meta-analysis. Subgroup analyses will be undertaken where appropriate for example where geographic location of studies varies, or study methodology may be significant to the quality of any primary analysis. Synthesis of qualitative findings will categorise themes extracted from primary studies based on the research question and will be described in narrative and schematic form. NVIVO (QSR International) software will assist inductive thematic synthesis.

Integration will follow a parallel results-based approach and will be configured via juxtaposition of synthesized quantitative results and synthesized qualitative findings framed around the JBI methodology. This approach will permit formal consideration of how or if the findings of one single method complements the other or can otherwise explore, contextualise or explain findings in the other results set.

**Statistics:** Statistical analysis will be undertaken using Stata (StataCorp) software with meta-analysis of primary and secondary quantitative outcomes, and subgroup analysis by intervention type, if data permits. Heterogeneity in the study methodologies is likely and so a random effects statistical model will be used for analysis. Statistical heterogeneity across study results will be estimated using the *I* ^2^ statistic and Chi ^2^ test. If meta-analysis is not possible, a narrative meta-synthesis will proceed.

**PPI:** The members of a Public and Patient Involvement (PPI) panel with experience in stroke will be involved in an advisory role.

## Discussion

Acute stroke care requires early patient assessment and care decisions within narrow time windows. People present with heterogeneous symptoms at variable time points from symptom onset. Telemedicine may offer the potential for early differentiation of a patient’s acute care needs through high quality consistent and responsive communication channels established between EMS and designated acute hospital sites. Mobile Stroke Units with on board imaging and point of care blood testing can facilitate rapid treatment but their cost can be prohibitive in many jurisdictions. Telephone, audio-visual and televisual communication methods aim to improve time to decision-making and time to treatment and may be more appropriate in resource poor or geographically remote settings. Formal and reliable communications strategies have the potential to account for deficiencies in local availability of expert opinion and decision-making, local health area resourcing or performance, and local geographic challenges such as distance from PSC or CSC or even prevailing travel conditions. This review will summarise and synthesise the evidence for a range of telecommunication-enabled interventions for triage in the pre-hospital acute care pathway that aim to improve patient outcomes, treatment rates and quality-associated time metrics through integrated care delivery networks. Additionally, the review will consider the context of effectiveness so that we might understand not only what is effective but also how those that experience or implement an intervention can impact or explain its effectiveness, feasibility and generalisability. While this review focuses on the use of telemedicine as a means of process improvement in acute stroke care provision, the results of this review will be interpreted in the context of recent broader emphasis on improving knowledge of and accessibility to IVT, EVT and stroke unit care which could contribute a confounding bias to the results. We aim that this review will help to inform the future research agenda in this field.

## Data Availability

No data are associated with this article. Zenodo: PRISMA-P checklist for “The impact of telemedicine enabled pre-hospital triage in acute stroke – a protocol for a mixed methods systematic review”, https://doi.org/10.5281/zenodo.6473361 ^15^. Data are available under the terms of the Creative Commons Attribution 4.0 International license (CC-BY 4.0).
