# Supplementary material for: The impact of telemedicine enabled pre-hospital triage in acute stroke – a protocol for a mixed methods systematic review
Source: HRB Open Res. 2023 Oct 30;5:32. Originally published 2022 Apr 28. [Version 2] doi: 10.12688/hrbopenres.13514.2 (PMC10632592; doi:10.12688/hrbopenres.13514.2)
Supplement: Peer review history

# Reviewer report: Reviewer response for version 2

**DOI:** 10.21956/hrbopenres.15138.r36838

**Published:** 2023-11-08

**Authors:** Philip M C Choi, Edward Callaly

**Affiliations:** 1 Eastern Health, Box Hill, Victoria, Australia; 2 Monash University, Clayton, Victoria, Australia

We had a good look at the revisions and comments by the authors. We are happy to recommend “Approved” status. We thank for the authors for addressing our comments.

Is the study design appropriate for the research question?

Yes

Is the rationale for, and objectives of, the study clearly described?

Yes

Are sufficient details of the methods provided to allow replication by others?

Partly

Are the datasets clearly presented in a useable and accessible format?

Not applicable

Reviewer Expertise:

Stroke and stroke telemedicine

We confirm that we have read this submission and believe that we have an appropriate level of expertise to confirm that it is of an acceptable scientific standard.

# Reviewer report: Reviewer response for version 1

**DOI:** 10.21956/hrbopenres.14745.r34911

**Published:** 2023-07-17

**Authors:** Philip M C Choi, Edward Callaly

**Affiliations:** 1 Eastern Health, Box Hill, Victoria, Australia; 2 Monash University, Clayton, Victoria, Australia

This comprehensive protocol, delineating the objectives, methodology, and anticipated outcomes in a significant and relevant field. Its integration of quantitative and ancillary qualitative analyses provides insightful exploration of the pre-hospital triage processes facilitated by telemedicine in acute stroke care. The methodological approach is robust, encompassing a thorough search strategy - including commitment to translating non-English articles - data extraction, and data synthesis procedures. The systematic review design exhibits considerable rigor, utilising recognised tools for evidence quality assessment. The points raised below are minor suggestions to an overall excellent protocol paper.

 The protocol refers to mobile stroke units within the context of stroke triage. Could there be a clearer distinction whether this review primarily focuses on telemedicine triage services, as implied in the title, or does it also extend to more interventional services? There is a broad spectrum of stroke telemedicine models ranging from paramedic-accessible triage hotlines to a live video link from a mobile stroke unit with a scanner onboard, connecting a stroke specialist who can directly assess patients, with the advantage of immediate imaging. Will models relying solely on telephone communication be classified as "telemedicine" within the context of this review? Furthermore, some services allow for remote treatment options such as regional thrombolysis, often referred to as the "drip and ship" model, or mobile stroke unit models. It would be beneficial to clarify these aspects for more comprehensive understanding.

 Pre-hospital triage is highly influenced by the use of large vessel occlusion scales. The final article would benefit from a discussion of these with consideration of how these impact the stroke telemedicine process.

 The protocol does not address how it will consider geographical variations in the availability and recognition of telemedicine. Such variations could influence the results, given that different regions may have distinct infrastructures, resources, and policy regulations, which could significantly affect the use and effectiveness of telemedicine for stroke triage. Consider, for instance, the implementation of perfusion imaging, extended thrombolysis criteria, endovascular clot retrieval availability, and ambulance service efficiency. These factors may cause substantial inter-regional variability in several key metrics.

 We concur that evaluating long-term functional outcomes is pivotal in assessing the effectiveness of these services. We observe that many studies on stroke telemedicine and mobile stroke units focus on process measures such as time to thrombolysis or time to clot retrieval. While these studies report encouraging results, there are fewer studies directly examining long-term disability outcomes, with some presenting mixed results. Your inclusion of 90-day mRS scores and other secondary outcomes should help elucidate these benefits.

 You state that secondary quantitative outcomes will encompass “onset to decision time thrombolysis” and “onset to decision time endovascular thrombectomy”. Do you mean the time difference between symptom onset and the decision to administer thrombolysis or thrombectomy? As this terminology does not appear to be universally documented, it may benefit from a clearer definition in the final article. The time when the decision for treatment or transfer is made is different from when the treatment or transfer happen. Door to needle time (door to treatment administration time), Door-in-door-out time (in cases where a primary stroke centre transfer out a patient with large vessel occlusion for consideration of thrombectomy), and Door to puncture time are the metrics most commonly recorded. Of course one should be able to replace “door” with “symptoms onset” time, the latter is fairly consistently reported as well. It is also stated in the “intervention” section of the quantitative inclusion criteria that “diagnosis of stroke at discharge must be confirmed”. Does this mean that all included patients must have reported evidence of stroke identified on imaging? We anticipate that many of the stroke telemedicine papers will report metrics such as door to needle time without necessarily definitively excluding patients with stroke mimics from these metrics. It would be worth mentioning how this challenge will be dealt with.

 It's worth acknowledging the significant global advancements in acute stroke medicine concerning training, infrastructure, and process implementation, all of which contribute to enhancing the speed and precision of acute stroke assessment and management. These developments, often introduced in conjunction with telemedicine services, could confound the impact of stroke telemedicine triage services in some study designs. A description of how this confounding bias will be mitigated could be beneficial.

 You've noted that funnel plots will be used to evaluate potential publication bias. In light of this, do you have plans to incorporate unpublished data into this review? We recognise that acquiring such data may pose challenges. In the data extraction section, you might want to correct the typographical error where the "s" in "study design" is mistakenly bolded.

 Despite the need for these clarifications, this paper presents a thorough systematic review protocol that would be a useful addition to the field.

Is the study design appropriate for the research question?

Yes

Is the rationale for, and objectives of, the study clearly described?

Yes

Are sufficient details of the methods provided to allow replication by others?

Partly

Are the datasets clearly presented in a useable and accessible format?

Not applicable

Reviewer Expertise:

Stroke and stroke telemedicine

We confirm that we have read this submission and believe that we have an appropriate level of expertise to confirm that it is of an acceptable scientific standard, however we have significant reservations, as outlined above.

# Response

**Published:** 2023-10-24

**Authors:** Deirdre McCartan

**Affiliations:** RCSI, Ireland

Dear Dr. Choi and Dr. Callaly,

 So many thanks to you both for taking the time to read and critically appraise the protocol for my mixed methods systematic review. Your comments and suggestions are both interesting and important and will strengthen the quality of the review.

 I have re-drafted my protocol to incorporate your feedback which should be available on the HRB site shortly I believe. The following are some points of note:

 In the “intervention” section of the inclusion criteria I have been more specific on the nature of the telemedicine-based triage that I am reviewing. It was not clear that the telephone triage should also have an objective clinical assessment tool to guide the consultation between the pre-hospital EMS crews and the hospital team. I will include a discussion of triage tools and their utility in the review.

 Additionally I agree that MSUs can be considered an intervention tool. I feel their role is also field-based triage of those who may benefit from EVT by potentially, depending on the time of onset if known, permitting rapid triage to a CSC rather than PSC.

 I have clarified also that I am interested only in pre-hospital triage and not in the hospital-based hub-and-spoke telemedicine model or the drip-and-ship model.

 Where geographic or inter-regional variation exists between studies I will undertake subgroup analysis to assess the impact of these differences.

 It became clear that my intention to only include confirmed stroke would outrule a number of useful and innovative studies that were interested in improving the pre-hospital pathway and in turn improving onset- to-door or door-to CT-times and so included all screened or evaluated patients. You intuitively predicted this and I appreciate this helpful point.

 I agree that onset-to-treatment decision time is not universally documented. It is a variable collected in the Irish National Audit of Stroke and is helpful in identifying transportation delays between a patient being accepted by a CSC and actually arriving for care, whether that be due to geography, ambulance availability, crew availability, prevailing weather or road conditions. I had thought that perhaps others would have incorporated this metric into their studies. I wish to include it as a noteworthy (but potentially absent) variable.

 I have acknowledged in the protocol discussion that improvements across the spectrum of stroke care is having a “raising all boats” effect in stroke and I will discuss this within the body of the review in order to contextualise findings and identify potential confounding effects.

 I have fixed the typo mistakenly bolded.

 I will publish unpublished data if it is made available to me.

 Again, it really is with thanks that I respond to your very detailed and thoughtful review of my protocol. I know that time demands are high and every increasing so your time on this is immensely helpful and appreciated.

 Deirdre

# Reviewer report: Reviewer response for version 1

**DOI:** 10.21956/hrbopenres.14745.r32200

**Published:** 2022-06-06

**Authors:** Jessalyn Holodinsky

**Affiliations:** 1 Department of Clinical Neurosciences, Cumming School of Medicine, University of Calgary, Calgary, AB, Canada

The authors have provided a thorough and comprehensive protocol for a mixed methods systematic review and potential meta-analysis of the impact of telemedicine for pre-hospital triage in acute stroke. This is a timely and important topic where a literature synthesis - especially one considering qualitative aspects - is lacking. 

 The study protocol is clear and would allow for easy replication. Appropriate statistical considerations for the quantitative analysis have been taken.

 I have only two suggestions: 

 1) For the qualitative analysis I'd ask the authors to clarify if themes will be inductively or deductively determined. 

 2) While it is stated that studies of low methodological quality will be noted but not excluded, I recommend performing sensitivity analyses excluding any studies of low methodological quality to ascertain if they exert any undue influence on estimates. 

 I look forward to the results of this mixed methods systematic review and meta-analysis.

Is the study design appropriate for the research question?

Yes

Is the rationale for, and objectives of, the study clearly described?

Yes

Are sufficient details of the methods provided to allow replication by others?

Yes

Are the datasets clearly presented in a useable and accessible format?

Not applicable

Reviewer Expertise:

Stroke epidemiology and biostatistics

I confirm that I have read this submission and believe that I have an appropriate level of expertise to confirm that it is of an acceptable scientific standard.
